# The Second-Meal Effect Under Free-Living Conditions: A Continuous Glucose Monitoring Study of Meal History and Postprandial Glycemia

**DOI:** 10.3390/nu18213697

**Published:** 2026-11-07

**Authors:** Thu Trang La, Jennell Jia Ying Koh, Wen Wei Ang, Jing Xuan Ng, Karishma Chandramohan Amudha, Jessie Jun Min Lim, Diana-Lyn Baptiste, Mei Hui Liu, Asim Shabbir, Roger Foo, Su Lin Lim, Kee Yuan Ngiam, Han Shi Jocelyn Chew

**Affiliations:** 1 Alice Lee Centre for Nursing Studies, Yong Loo Lin School of Medicine, National University of Singapore, Level 5, Centre for Translational Medicine, Block MD6, 14 Medical Drive, Singapore 117599, Singapore; trang.la@nus.edu.sg (T.T.L.);; 2 School of Nursing, Johns Hopkins University, Baltimore, MD 21205, USA; 3 Department of Food Science and Technology, National University of Singapore, Singapore 117542, Singapore; 4 Department of Surgery, National University Hospital, Singapore 119074, Singapore; 5 Department of Cardiology, National University Hospital, Singapore 119074, Singapore; 6 Yong Loo Lin School of Medicine, National University of Singapore, Singapore 119228, Singapore; 7 Asia Longevity Medical Centre, Singapore 238859, Singapore; 8 Department of Biomedical Informatics, Yong Loo Lin School of Medicine, National University of Singapore, Singapore 119228, Singapore; 9 Centre for Behavioural and Implementation Science Interventions, Yong Loo Lin School of Medicine, National University of Singapore, Singapore 117597, Singapore

**Keywords:** continuous glucose monitoring, postprandial glycemia, second-meal effect, free-living conditions, within-person analysis, diet

## Abstract

**Background/Objectives**: Excess adiposity is associated with abnormal postprandial glucose responses (PPGRs), and controlled studies show that one meal can influence the PPGR of the next. Whether this carry-over occurs in everyday eating is unclear. We examined whether the timing, composition, and PPGR of the preceding meal were associated with the subsequent PPGR. **Methods**: This observational secondary analysis included 55 Southeast Asian adults with excess body weight and 342 within-day consecutive eating-occasion pairs recorded using a smartphone app and continuous glucose monitoring. Participant fixed-effects linear regression examined 12 preceding-meal characteristics, including intermeal interval (IMI), glycemic load, total sugar, protein, and saturated fat. The outcome was the log-transformed positive incremental area under the glucose curve ln(1 + positive 0–120 min iAUC after the subsequent meal. Each characteristic was examined separately per within-person standard deviation (SD), adjusting for current-meal characteristics, premeal glucose, and temporal factors. Confidence intervals and p values used 2000 participant-cluster bootstrap resamples; Benjamini–Hochberg q values addressed the 12 tests. **Results**: Higher preceding-meal protein was associated with lower iAUC (−13.9%; 95% confidence interval (CI), −23.1 to −4.2; *p* = 0.008; q = 0.032), whereas total sugar was associated with higher iAUC (+29.6%; 95% CI, +16.9 to +45.2; *p* = 0.001; q = 0.006). A 1 SD (1.9 h) longer IMI was associated with 29.1% higher iAUC (95% CI, 15.0 to 48.8; *p* = 0.001; q = 0.006). The saturated-fat estimate was uncertain (+14.8%; 95% CI, −0.8 to +35.2; *p* = 0.066; q = 0.198). **Conclusions**: Preceding-meal sugar, IMI and protein were associated with subsequent glucose responses. Protein was sensitive to additional macronutrient adjustment; saturated fat remained uncertain. Incomplete metabolic and dietary information limits causal interpretation.

## 1. Introduction

Overweight and obesity have become major global drivers of cardiometabolic disease. In 2022, an estimated 2.5 billion adults worldwide were overweight, including more than 890 million living with obesity, and the global prevalence of adult obesity had more than doubled since 1990 [1,2]. Excess adiposity increases the risk of type 2 diabetes, cardiovascular disease, and metabolic dysfunction even before overt diabetes develops. Excess adiposity promotes insulin resistance and impaired glucose regulation, such that adults with overweight may already exhibit reduced insulin sensitivity and abnormal postprandial glucose responses (PPGRs) despite having normal fasting glucose levels [3,4]. This question is especially important in Asian populations, who develop type 2 diabetes at younger ages and at lower BMI than White populations [5,6,7]. Consequently, clinically relevant metabolic dysfunction may occur at modest levels of adiposity, making postprandial glucose regulation an especially important marker of early metabolic risk in Asian populations.

However, the PPGR to a meal is not determined solely by the meal itself. Meal patterning, including intermeal interval (IMI) and preceding-meal composition, has been shown to influence the subsequent meal’s PPGR [8,9,10]. This carry-over phenomenon is commonly described as the second-meal effect [11]. Controlled feeding studies have demonstrated this effect. A low-glycemic-index dinner reduced the glycemic response to breakfast the following morning, while high-protein breakfasts modified post-lunch insulin and incretin responses and, in healthy adults, reduced glucose exposure after later meals [8,9,10].

However, most of this evidence comes from controlled feeding studies [8,11,12,13,14,15,16]. These studies typically examine standardized meals at predefined times, whereas free-living eating comprises irregular sequences of meals and snacks that may not conform to conventional meal patterns. Hence, much less is known about whether comparable carry-over effects operate across consecutive eating occasions in everyday life. Free-living studies using continuous glucose monitoring show that PPGRs vary substantially within individuals and are associated with meal composition, time of day, physical activity, and sleep [17]. This gap is particularly relevant in Asian populations, where rice is a major contributor to dietary glycemic load and postprandial responses show substantial interindividual and ethnic variation [18,19]. We therefore examined whether the timing, composition, and glycemic response of the immediately preceding eating occasion were associated with positive incremental area under the glucose curve over 0–120 min following the subsequent eating occasion in adults with overweight in Singapore.

## 2. Materials and Methods

### 2.1. Study Design and Participants

As part of a larger three-arm randomized controlled trial that tested the effectiveness of using the eating Trigger Response Inhibition Program (eTRIP) app and CGM in promoting weight loss among adults with excess body weight, the present analysis included participants from the eTRIP intervention arm who underwent 10 days of monitoring with a Dexcom G6 CGM sensor (Dexcom, Inc., San Diego, CA, USA) [20]. Participants were recruited between 22 January 2025 and 20 January 2026 at the National University of Singapore.

Participants could view their CGM readings during monitoring. Food choices and eating occasions remained participant-selected during this free-living educational and behavioral intervention. No dietary plan was provided for participants.

The parent trial’s registered inclusion criteria were age 21–65 years, body mass index (BMI) ≥ 23 kg/m^2^, ability to comprehend English, use of a smartphone capable of downloading apps, willingness to wear a CGM sensor and travel to the National University of Singapore, and ability to provide informed consent. Registered exclusion criteria were use or intended use of other weight-loss apps, pregnancy or lactation, diagnosed severe mental disorders, prescribed meal replacements or pharmacotherapy, serious medical conditions, and surgery scheduled within the next six months.

The trial was approved by the National University of Singapore Institutional Review Board (NUS-IRB-2023-597). The trial was registered retrospectively (ClinicalTrials.gov NCT07395466; first posted 9 February 2026) after enrolment began on 22 January 2025, owing to an administrative oversight. The present analysis is observational and was not pre-specified in the registration. This study is reported in accordance with the STROBE-nut extension [21] (Checklist S1).

**Figure 1:**
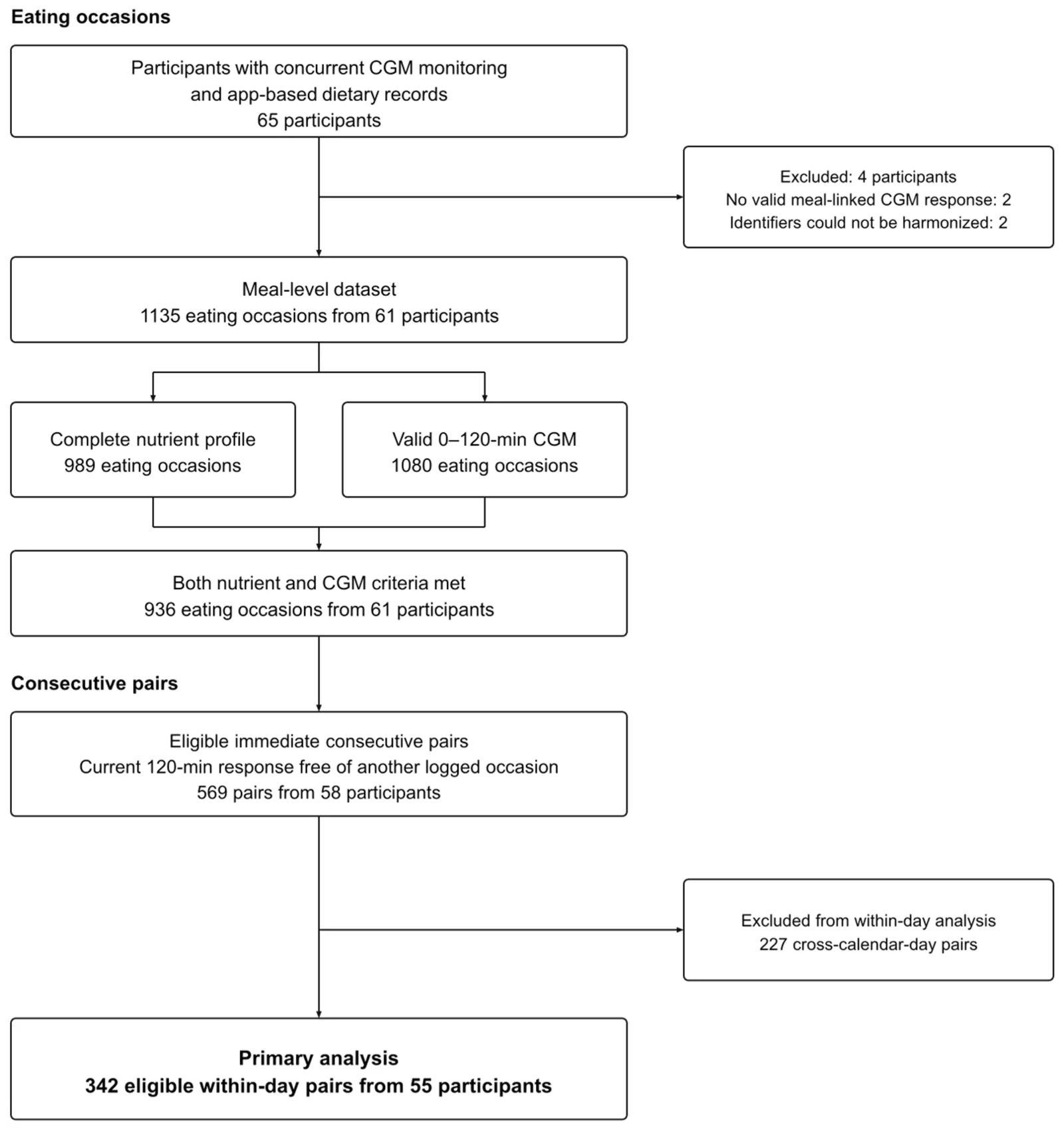
Derivation of the primary analytic cohort of 55 participants and 342 within-day pairs. The exclusion of two participants with self-reported diabetes is a separate sensitivity analysis (Table S4). CGM, continuous glucose monitoring.

Among 65 participants who received a CGM sensor and logged foods using eTRIP’s image-recognition food-logging feature, 61 participants contributed at least one valid meal-linked CGM window with 1135 recorded eating occasions; 55 contributed at least 1 eligible consecutive pair to the present analysis (Figure 1).

### 2.2. Baseline Characteristics

Data collected on participants included age, sex, race, anthropometry, body composition, physical activity, eating behavior, and psychological symptoms. Height was measured using a wall-mounted measuring tape. Weight, body fat percentage, fat mass, skeletal muscle mass, and visceral fat level were assessed using an InBody 120 body-composition analyzer (InBody Co., Ltd., Seoul, Republic of Korea). Physical activity was classified as low, moderate, or high using the self-recorded International Physical Activity Questionnaire–Short Form (IPAQ-SF). Eating behavior was assessed using the Three-Factor Eating Questionnaire (TFEQ) comprising three subscales, namely uncontrolled eating, cognitive restraint, and emotional eating. Self-regulation of eating behavior was assessed using the Self-Regulation of Eating Behaviour Questionnaire (SREBQ). Anxiety and depression were assessed using the 2-item Generalized Anxiety Disorder (GAD-2) and 2-item Patient Health Questionnaire (PHQ-2), respectively [20,22].

### 2.3. Dietary Records and Nutrient Processing

Participants used the eTRIP mobile application to photograph and describe the foods and beverages that they consumed. The app’s image-recognition workflow proposed candidate food labels for participants to review and confirm. Dietary supplements were not recorded or included in the nutrient estimates. Logged food names were matched to the Singapore Food Insights Database of the Health Promotion Board (HPB) [23]. Of 1135 eating episodes, 989 had nutrient data matched to HPB’s nutrition database and were eligible for nutrient analyses. Food-specific glycemic index (GI) values were assigned to each meal based on the Singapore Food Insights Database and, if unavailable, from the 2021 International Tables of Glycemic Index [23,24]. Meal glycemic load (GL) was calculated as the sum of food GI multiplied by available carbohydrate and divided by 100; weighted GI was the carbohydrate-weighted mean of component-food GI values [25]. Twelve preceding-meal factors were analyzed, namely IMI, meal duration, energy, GL, iAUC, weighted GI, total sugar percentage of energy, fiber density, carbohydrate percentage of energy, saturated fat percentage of energy, total fat percentage of energy, and protein percentage of energy [16,26,27,28].

A valid food-log day contained at least two retained nutrient-complete occasions; this does not establish complete daily intake.

### 2.4. CGM Outcome

Participants wore a Dexcom G6 CGM sensor that recorded interstitial glucose at 5 min intervals. PPGR was examined in the window between 30 min before and 120 min after a self-reported eating episode; positive incremental area under the curve (iAUC) was calculated from meal onset to 120 min postprandially using the trapezoidal rule, with the median glucose concentration during the 30 min premeal period as the baseline and glucose values below baseline assigned an incremental value of zero [29]. We transformed iAUC as ln(1 + iAUC) for analysis.

Participants received app alerts for high CGM glucose readings; no further study action was taken. Glucose was assessed only by CGM, without capillary blood glucose confirmation. Use of glucose-lowering medication was not recorded.

### 2.5. Construction of Consecutive Eating-Occasion Pairs

Each eating episode, including snacks, was linked to the preceding one on the same calendar date (00:00–23:59). Eligible pairs required nutrient-complete records for both occasions, a valid current-meal CGM response and a positive start-to-start interval. Pairs were excluded when another eating occasion occurred during the current occasion’s 0–120 min response window because its iAUC could not be attributed to the current occasion alone. The final dataset comprised 342 non-overlapping pairs from 55 participants (Figure 1).

### 2.6. Statistical Analysis

Participant characteristics and study variables were summarized as mean ± SD, median (IQR) or counts (%), as appropriate. Collinearity was assessed using variance inflation factors (VIFs). Each preceding-meal factor was evaluated separately using participant fixed-effects linear regression.

Continuous exposures were person-mean-centered and scaled to one factor-specific within-person SD. All primary models adjusted for current-meal carbohydrate intake (g), current-meal energy intake, premeal glucose, study day, clock hour and weekend status. Models of exposures other than IMI additionally adjusted for log-transformed IMI. The IMI model included preceding-meal energy intake, protein and fat percentages, and GL. The meal-duration and energy models included preceding-meal protein and fat percentages; the duration model additionally included preceding-meal energy intake. Models of preceding-meal GL and carbohydrate, fat and protein percentages included preceding-meal energy intake. The sugar model included preceding-meal energy intake and protein and fat percentages; the saturated-fat model included preceding-meal energy intake and total fat percentage; the fiber-density model included preceding-meal energy intake and GL; the weighted-GI model included preceding-meal energy and carbohydrate intake; and the preceding-iAUC model included preceding-meal energy intake, GL, and protein and fat percentages. Missing covariates were handled by complete-case analysis without imputation.

The outcome was transformed as ln(1 + positive iAUC). Exponentiated coefficients describe percentage differences in iAUC + 1 per within-person SD increase in the exposure. Confidence intervals and two-sided sign-tail p values used 2000 participant-cluster bootstrap resamples. Benjamini–Hochberg q values were calculated across the 12 primary exposure tests. IMI-by-composition interactions were examined as exploratory analyses (Table S1).

Sensitivity analyses additionally adjusted for current-meal protein and total fat (% energy) and fiber density (Table S3), excluded participants with self-reported diabetes (Table S4), excluded suspected unlogged eating events identified from intermeal CGM (Table S6), and adjusted for eating context (Table S7). Exploratory analyses examined liquid and solid sugar sources (Table S8).

External consistency was examined using the independently processed CGMacros dataset for associations of next-meal iAUC with IMI, preceding-meal protein, fat and carbohydrate percentages of energy, and fiber density. Effect estimates were expressed per within-person SD of the exposure. Given differences in food mapping, exposure scaling, and covariate availability between datasets, the analyses were interpreted as evidence of directional consistency rather than independent validation [30].

For illustration, we contrasted naturally occurring low- vs. high-sugar preceding meals (extremes of the observed distribution) within the same person across three sequences (breakfast to lunch, lunch to dinner, and snack to dinner), selecting the closest match on current-meal energy and premeal baseline glucose.

Analyses used R 4.5.0 and Python 3.14.2. All tests were two-sided. Within the 12-exposure family, primary associations with q < 0.05 were considered supported after false discovery rate (FDR) correction.

## 3. Results

### 3.1. Participants and Analytic Eating-Occasion Pairs

The analytic sample included 55 participants (median age, 27 years; 29 women [52.7%]; median BMI, 26.4 kg/m^2^) who contributed 342 eligible consecutive pairs occurring within the same calendar day. Two participants (3.6%) self-reported diabetes (Table 1). Participants contributed a median of five pairs (IQR, 3–9). The most frequent eating occasion was dinner (178 pairs [52.0%]), followed by lunch (89 [26.0%]), snack (66 [19.3%]), and breakfast (9 [2.6%]). The most frequent transitions were lunch to dinner (*n* = 110), breakfast to lunch (*n* = 79), and snack to dinner (*n* = 53) (Table 1 and Table 2).

### 3.2. Relationship Between Preceding-Meal Factors and PPGR

**Figure 2:**
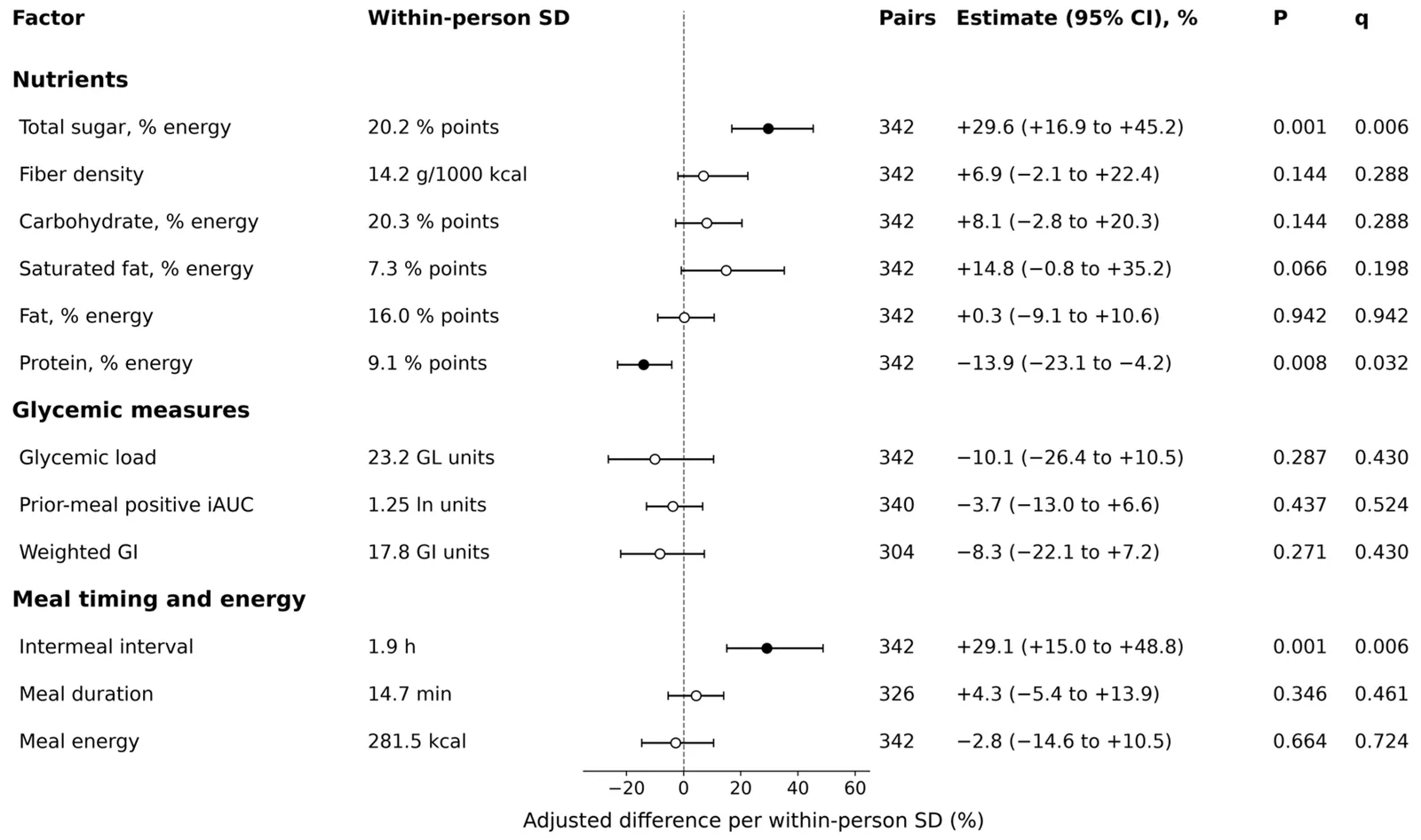
Associations of preceding-meal factors with next-meal positive 0–120 min iAUC. Primary models adjusted for current-meal carbohydrate grams, energy intake, premeal glucose, study day, clock hour and weekend status. Non-IMI factors additionally adjusted for log-transformed IMI. Exposure-specific preceding covariates are described in the Section 2.6; Table S3 reports additional current-meal macronutrient adjustment. Effects are percentage differences in iAUC + 1 per within-person SD. Intervals and p values use 2000 participant-cluster bootstrap resamples. q values use Benjamini–Hochberg correction across 12 tests; filled markers indicate q < 0.05. CI, confidence interval; GI, glycemic index; iAUC, incremental area under the curve; SD, standard deviation.

**Figure 3:**
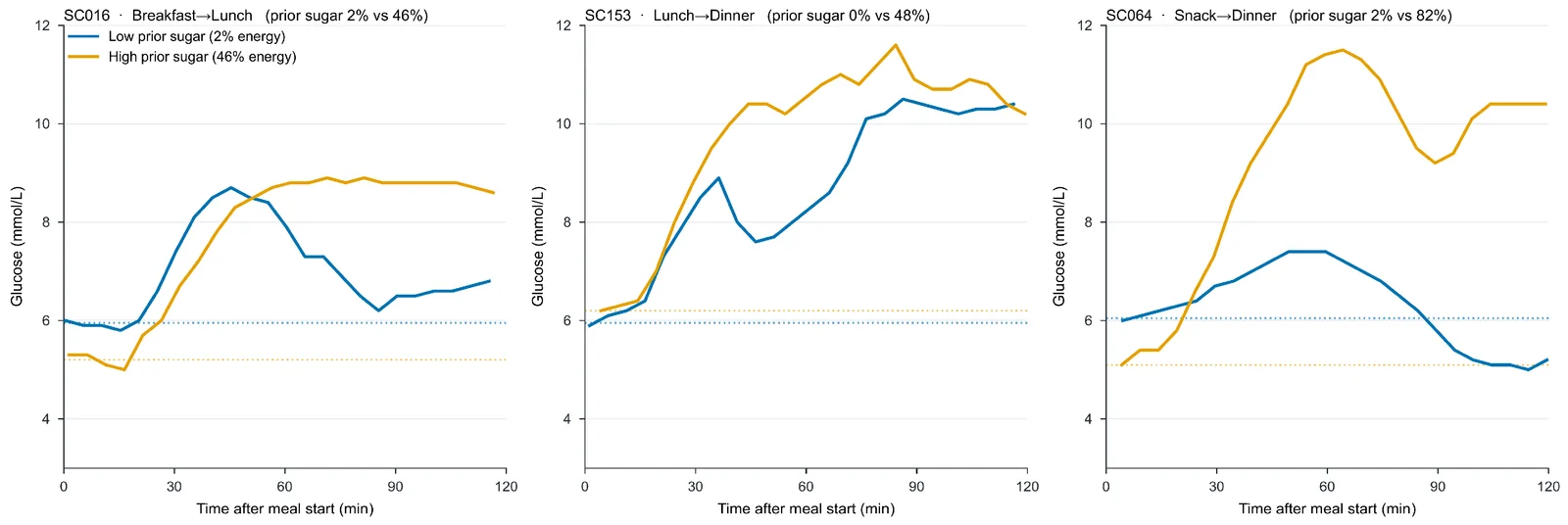
Within-participant CGM profiles after eating occasions preceded by lower- versus higher-sugar meals. Three participant examples were selected for visualization based on the closest match on current-meal energy and premeal glucose baseline. Solid lines show glucose concentrations over 0–120 min following the current eating occasion; dotted lines indicate the corresponding premeal glucose baselines. Percentages in parentheses indicate total sugar as a percentage of energy in the preceding eating occasion.

As shown in Figure 2, higher preceding-meal total sugar (% energy) was associated with higher next-meal iAUC (+29.6%; 95% CI, +16.9 to +45.2; *p* = 0.001; q = 0.006). Figure 3 provides illustrative within-participant CGM profiles; these selected examples are not independent tests of a sugar effect.

Higher preceding-meal protein (% energy) was inversely associated with next-meal iAUC (−13.9%; 95% CI, −23.1 to −4.2; *p* = 0.008; q = 0.032). The saturated-fat estimate was positive (+14.8%; 95% CI, −0.8 to +35.2; *p* = 0.066; q = 0.198) but did not meet the FDR threshold.

A one SD (1.9 h) longer IMI was associated with higher next-meal iAUC (+29.1%; 95% CI, +15.0 to +48.8; *p* = 0.001; q = 0.006).

The remaining eight factors—meal duration, energy, glycemic load, prior-meal iAUC, weighted GI, fiber density, carbohydrate (% energy), and total fat (% energy)—were not significantly associated with next-meal iAUC (Figure 2).

No interaction between IMI and preceding-meal composition was significant (all *p* > 0.17; Table S1). VIF diagnostics did not indicate severe multicollinearity (Table S5).

### 3.3. Sensitivity Analyses

IMI and sugar associations persisted after additional adjustment for current-meal macronutrients or eating context and exclusion of participants with self-reported diabetes or CGM-flagged pairs (Tables S3, S4, S6 and S7). Protein did not meet the FDR threshold after macronutrient adjustment, and saturated fat remained unsupported after correction for multiple testing. Both liquid and solid sugar sources showed positive associations (Table S8).

### 3.4. Exploratory External Comparison

External consistency was examined using the independently processed CGMacros dataset. In CGMacros, IMI was positively associated with next-meal iAUC (+33.6% per 1.15 h increase; 615 pairs from 45 participants; 95% CI, +13.3 to +54.7; *p* = 0.001). The preceding-meal protein estimate was inverse but imprecise (−10.3% per within-person SD; 95% CI, −18.5 to +3.3; *p* = 0.166). These findings indicate directional consistency rather than independent validation (Table S2).

## 4. Discussion

In this exploratory secondary analysis of 342 within-day pairs from 55 participants, longer IMI, higher preceding-meal sugar share and lower preceding-meal protein share were associated with higher next-meal iAUC. IMI and total sugar showed the most consistent associations in the primary and macronutrient-adjusted models, including after correction for multiple testing.

### 4.1. Preceding-Meal Protein

The inverse protein association is supported most directly by a randomized crossover study in which a high-protein breakfast lowered the glucose response to white bread eaten 4 h later [16]. Other results are supportive but less consistent. In adults with type 2 diabetes, a high-protein breakfast increased insulin and glucose-dependent insulinotropic polypeptide responses to lunch 4 h later [15]. In another trial, both a whey-enriched breakfast and a carbohydrate-rich breakfast reduced the insulin response to lunch compared with fasting; the study did not show a clear protein-specific benefit after lunch [9]. The effect may therefore reflect protein, breakfast consumption, or both. Protein can stimulate insulin and incretin secretion and slow gastric emptying, although these effects vary by dose, timing, and metabolic status [31]. A meta-analysis of 16 crossover trials found that whey protein taken shortly before a meal reduced postprandial glucose [32], although this preload evidence cannot be applied directly to ordinary mixed meals eaten several hours earlier.

### 4.2. Preceding-Meal Sugar and Saturated Fat

The association with preceding-meal sugar was not accompanied by similar associations for glycemic index, glycemic load, carbohydrate percentage, or preceding-meal iAUC. This suggests that the finding may not reflect a general effect of carbohydrate quantity or glycemic quality. Total sugar may instead capture other features of the preceding meal, but these could not be distinguished in the present analysis because added and naturally occurring sugars were not separated.

Fructose metabolism in the liver may affect subsequent glucose regulation without a corresponding rise in the immediate postprandial response. A small randomized crossover trial reported lower hepatic insulin sensitivity after three weeks of fructose-sweetened beverages than after glucose-sweetened beverages, despite similar fasting glucose [33]. Whether a single mixed meal produces a similar effect remains unclear. We did not measure fructose intake, hepatic glucose production or insulin sensitivity. Uncertainty in food classification and nutrient estimates also limits comparisons between liquid and solid sugar sources.

The saturated-fat estimate was positive, but its confidence interval included zero and it did not meet the primary FDR threshold. Experimental studies suggest that the fat content of a preceding meal can influence the glycemic response to a subsequent meal. In a crossover study, a relatively high-fat meal increased the glucose response to a subsequent high-carbohydrate meal, while earlier two-meal studies also demonstrated carry-over effects of meal fat on subsequent glucose and lipid responses [28,34]. However, these studies examined dietary fat more broadly and do not establish a specific effect of saturated fat. Evidence comparing saturated and unsaturated fats on glucose–insulin homeostasis is also inconsistent [35].

Related evidence comes from studies of food order within a meal. Eating carbohydrate last reduced glucose and insulin excursions in adults with type 2 diabetes [36]. The PATTERN crossover study also showed that macronutrient order changed glucose, insulin, and incretin responses in adults without diabetes [37]. These studies show that the metabolic response to a meal depends partly on the sequence in which its components are consumed. Our study extends this idea across separate eating occasions. However, food order within one meal and carry-over between meals may involve different mechanisms.

### 4.3. Intermeal Interval

Our finding that a longer IMI was associated with a higher next-meal iAUC is consistent with studies showing that a longer fasting period can increase the glucose response to the subsequent meal. In a randomized crossover study of 26 healthy adults, delaying lunch by 2 h increased the post-lunch glucose peak, whereas the resulting shorter lunch-to-dinner interval was associated with a lower dinner glucose response [14]. Breakfast-omission studies provide further support. In adults with type 2 diabetes, post-lunch glucose was much smaller when breakfast had been eaten, and the pre-lunch free fatty acid concentration was positively related to the lunch response [13]. Another randomized crossover trial found that skipping breakfast worsened glycemia after both lunch and dinner, with lower insulin and glucagon-like peptide 1 responses [10]. Several mechanisms could contribute to this carry-over effect. The preceding meal may influence subsequent glucose regulation through changes in insulin sensitivity and hormonal responses. Suppression of circulating fatty acids may improve insulin action, while earlier insulin and incretin responses may limit the next glucose rise [10,13]. These pathways were not measured in our study and therefore remain speculative. A longer IMI may also mark a missed meal, different physical activity, or unrecorded intake.

This acute response should be distinguished from the longer-term metabolic effects of time-restricted eating. Studies of repeated time-restricted eating generally report improvements in insulin sensitivity and other metabolic outcomes, although findings vary by study design and population. In adults with type 2 diabetes, a 10 h eating window improved fasting glucose, 24 h glucose, and time in normoglycemia over 3 weeks, although insulin sensitivity did not improve [26]. Early time-restricted feeding also improved insulin sensitivity in a small trial of men with prediabetes [38]. In an acute study, however, extending the overnight fast from 12 to 16 h increased breakfast glucose iAUC by 37% in women, but not in men [39]. These findings address different time scales: a longer fasting interval may increase glucose exposure at the next meal, while repeated time-restricted eating may improve overall metabolic health over time. Our findings concern an association between the recorded interval and the subsequent glucose response and do not establish that longer fasting is metabolically harmful.

Time of day may also influence PPGR. Controlled studies have reported larger glycemic responses at dinner than at breakfast, with morning–evening differences that may vary by chronotype [40,41]. A recent free-living study of 514 adults also found that glycemic responses to meal glycemic load varied with meal timing and participant characteristics [42]. Our models adjusted for clock time, reducing the possibility that the interval result only reflected poorer glucose tolerance later in the day. Nevertheless, interval and clock time are related exposures and cannot be fully separated in an observational study.

### 4.4. Strengths and Limitations

The present study has several strengths. Consecutive, non-overlapping eating occasions allowed us to distinguish the preceding meal from the meal producing the outcome. Detailed diet records were linked to CGM under free-living conditions. The participant fixed-effects approach compared eating occasions within the same individual, thereby controlling for all measured and unmeasured participant characteristics that remained constant during monitoring. Most previous second-meal experiments used fixed test meals or preloads in small crossover samples. Our study extends controlled feeding research by examining participant-selected meals and snacks under free-living conditions.

The study also has limitations. The observational design precludes causal inference. Physical activity, sleep, stress, and other behaviors can change between meals and may confound the associations. Physical activity and sleep are related to free-living postprandial glucose, and insufficient sleep can worsen responses under standardized meal conditions [17,43]. Food records may contain errors in portion size, nutrient composition, or timing. An unrecorded snack could cause the wrong preceding occasion to be selected; more broadly, detecting eating events from CGM alone remains methodologically difficult. CGM measures interstitial rather than blood glucose and is subject to physiological lag and sensor error [44,45]. The 342 pairs limited precision, particularly because 12 exposures and multiple post hoc sensitivities were examined. The findings may not generalize beyond adults with overweight or obesity in Singapore. Agreement in direction with the external CGMacros analysis is encouraging, but it is not an independent replication because the cohorts and methods differed.

Metabolic screening information, including HbA1c, fasting plasma glucose (FPG) and biochemically confirmed diabetes/prediabetes status, was unavailable for this secondary analysis. Use of glucose-lowering medication was not recorded, and CGM readings were not confirmed by capillary blood glucose testing. The sensitivity analysis excluding the two participants with self-reported diabetes cannot rule out undiagnosed dysglycemia or medication-related confounding. Visible CGM feedback and the app may have changed food choices, meal timing, exercise and logging. The CGM heuristic is not a validated meal detector; physiological and sensor effects may cause rises, and short or poorly covered intervals were unevaluable. Context was self-reported with imperfect timing and no exercise dose, limiting cohort-wide adjustment. Overnight pairs were excluded because sleep, fasting and morning physiology differ from within-day intervals, and the available data were insufficient to characterize these conditions reliably.

### 4.5. Implications

Nutritional epidemiology is built largely around what people eat: meals are typically treated as independent exposures, and our results suggest that the same food may carry a different glycemic consequence depending on the previous meal’s timing and composition. These considerations are directly relevant to CGM-based dietary phenotyping and precision nutrition. Efforts in glucotyping [46] and predicting individual postprandial responses from meal composition, the gut microbiome, and anthropometry have advanced rapidly [47]. Our findings suggest that features describing the preceding eating occasion are candidate predictors that can be derived at no additional measurement cost from data already collected in CGM–diet studies. Incorporating them may improve both phenotypic classification and response prediction. Studies of postprandial responses should consider the timing and composition of the preceding meal as potential confounders. Whether these associations are causal and clinically meaningful requires confirmation in randomized studies that independently manipulate IMI and preceding-meal composition while standardizing the subsequent meal.

## 5. Conclusions

In conclusion, both the interval and composition of the preceding meal were associated with the glucose response to the next eating occasion. The evidence was strongest for IMI and total sugar, while protein and saturated fat showed less consistent associations. These findings extend controlled second-meal research into daily life. Further research is warranted to investigate underlying biological and behavioral mechanisms influencing glycemic fluctuations. Replication should separately analyze sugar-sweetened beverages and solid foods and distinguish added or free sugars from intrinsic sugars. Such approaches may clarify the health implications of different sugar sources and inform future dietary recommendations.

## Figures and Tables

**Table 1:** Participant characteristics in the analytic sample.

Characteristic	Overall
**Demographics**	
Age, years	27.0 (23.0, 39.0)
Female	29 (52.7%)
Male	26 (47.3%)
Race	
Chinese	44 (80.0%)
Malay	3 (5.5%)
Indian	4 (7.3%)
Other	4 (7.3%)
Marital status	
Single	42 (76.4%)
Married	12 (21.8%)
Other	1 (1.8%)
Highest education level	
Secondary	0 (0.0%)
Diploma/polytechnic	23 (41.8%)
University	32 (58.2%)
Employment	
Not employed/other	2 (3.6%)
Employed	31 (56.4%)
Student	22 (40.0%)
Occupation (grouped)	
Student	22 (40.0%)
Professional/office/management	16 (29.1%)
Other occupation	7 (12.7%)
Health/social care	6 (10.9%)
Service/operations	4 (7.3%)
**Body composition**	
BMI, kg/m^2^	26.4 (24.9, 29.0)
Body fat, %	31.1 ± 7.8
Fat mass, kg	22.2 (18.9, 27.3)
Skeletal muscle mass, kg	30.1 ± 6.6
Visceral fat level	10.2 ± 4.1
Self-reported diabetes, No. (%)	2 (3.6)
**Baseline questionnaires**	
SREBQ total	2.9 ± 0.4
TFEQ uncontrolled eating	46.4 ± 16.8
TFEQ cognitive restraint	47.7 ± 17.0
TFEQ emotional eating	53.1 ± 24.2
PHQ-2	0.0 (0.0, 2.0)
GAD-2	2.0 ± 1.7
**Physical activity**	
IPAQ-SF: Low	16 (29.1%)
IPAQ-SF: Moderate	23 (41.8%)
IPAQ-SF: High	16 (29.1%)
**Diet/eating-pattern summary**	
Valid food-log days ^1^	5.5 ± 2.6
Eating occasions per valid day, events/day	2.5 (2.2, 2.7)
Carbohydrate, % energy	48.4 ± 7.9
Protein, % energy	16.5 (14.2, 18.5)
Fat, % energy	35.0 (31.5, 37.6)
Fiber density, g/1000 kcal	10.3 ± 4.1
Mean meal GI	52.2 (45.8, 57.6)
Mean meal GL	31.2 ± 10.3
Mean eating-window duration, h	8.3 (7.2, 9.7)
**CGM summary**	
Valid CGM days ^2^	9.0 (9.0, 9.0)
Sensor coverage, % ^3^	98.8 (91.9, 99.2)
Mean glucose, mg/dL (mmol/L)	113.5 (108.1, 122.5) [6.3 (6.0, 6.8)]
Glucose CV, %	18.5 ± 4.2
Time 70–140 mg/dL (3.9–7.8 mmol/L), %	90.3 (82.3, 93.0)
**Analytic eating-occasion pairs**	
Eligible pairs, No.	342
Pairs per participant	5 (3–9)
Participant-level mean iAUC, mg/dL·min (mmol/L·min)	2585.6 (1583.8–3747.7) [143.5 (87.9–208.0)]
**Current eating occasion, No. (%)**	
Breakfast	9 (2.6)
Lunch	89 (26.0)
Dinner	178 (52.0)
Snack	66 (19.3)
**Most frequent transitions, No.**	
Lunch → dinner	110
Breakfast → lunch	79
Snack → dinner	53
Lunch → snack	27
Dinner → snack	22
Other transitions	51

Values are No. (%), mean ± SD, or median (IQR), as appropriate. Diet summaries use nutrient-complete meals among the final 55 participants. Glucose values are shown in mg/dL with mmol/L in square brackets. Diabetes was self-reported and was not biochemically verified. ^1^ A valid food-log day contained at least two retained nutrient-complete occasions. This does not establish complete daily intake; selected-record daily-energy totals are omitted. ^2^ A valid CGM day required ≥202 of 288 expected 5 min readings. ^3^ Sensor coverage was calculated against 2880 planned readings and capped at 100%. BMI, body mass index; CGM, continuous glucose monitoring; CV, coefficient of variation; GAD-2, 2-item Generalized Anxiety Disorder scale; GI, glycemic index; GL, glycemic load; iAUC, incremental area under the curve; IPAQ-SF, International Physical Activity Questionnaire–Short Form; IQR, interquartile range; PHQ-2, 2-item Patient Health Questionnaire; SD, standard deviation; SREBQ, Self-Regulation of Eating Behaviour Questionnaire; TFEQ, Three-Factor Eating Questionnaire.

**Table 2:** Characteristics of the preceding and current eating occasions by sex.

Characteristic	Preceding Eating Occasion			Current Eating Occasion		
	Total (*n* = 342)	Male (*n* = 175)	Female (*n* = 167)	Total (*n* = 342)	Male (*n* = 175)	Female (*n* = 167)
**Meal timing and composition.**						
Intermeal interval, h	4.6 (3.3–6.5)	4.9 (3.5–6.5)	4.5 (3.1–6.5)	—	—	—
Energy, kcal	423.7 (168.2–696.8)	443.9 (173.0–734.8)	355.8 (167.6–675.6)	542.7 (232.3–789.3)	609.0 (313.9–780.5)	408.7 (210.0–789.2)
Carbohydrate, g	48.3 (20.9–80.7)	49.2 (21.0–80.6)	47.4 (21.1–79.9)	57.3 (23.1–85.1)	66.2 (26.9–86.3)	49.8 (18.9–80.8)
Carbohydrate, % energy	51.2 ± 22.3	50.1 ± 23.1	52.4 ± 21.5	44.2 ± 21.2	45.8 ± 22.4	42.6 ± 19.7
Protein, g	14.8 (4.2–32.9)	18.2 (4.6–34.5)	12.5 (4.1–30.8)	25.5 (7.4–34.9)	27.1 (10.5–35.3)	19.5 (6.2–34.9)
Protein, % energy	16.1 ± 11.2	17.1 ± 12.0	15.1 ± 10.1	18.1 ± 10.7	17.9 ± 10.0	18.4 ± 11.3
Fat, g	13.7 (4.3–29.0)	13.7 (4.4–32.1)	13.6 (4.2–26.9)	21.6 (7.7–35.3)	26.4 (8.9–37.5)	17.9 (6.9–30.1)
Fat, % energy	32.1 ± 17.2	32.1 ± 17.4	32.2 ± 17.0	37.5 ± 18.8	35.7 ± 17.9	39.4 ± 19.5
Fiber, g	2.4 (0.5–6.0)	2.4 (0.5–6.3)	2.3 (0.6–5.4)	4.1 (1.4–7.9)	5.1 (1.7–7.9)	3.3 (1.0–7.4)
Fiber density, g/1000 kcal	6.4 (2.4–11.5)	6.4 (2.3–11.8)	6.3 (2.4–11.4)	8.8 (4.1–12.0)	8.9 (4.6–12.0)	8.7 (3.1–12.0)
Glycemic load	24.3 (8.2–49.4)	27.2 (8.3–48.4)	23.5 (7.7–49.7)	31.9 (8.1–60.3)	37.1 (11.2–62.6)	26.5 (4.9–49.1)
Weighted GI	52.1 ± 23.7	52.1 ± 23.9	52.2 ± 23.6	52.2 ± 26.3	55.7 ± 25.9	48.6 ± 26.4
**Eating context, No. (%)**						
Reported hunger	86 (25.1)	44 (25.1)	42 (25.1)	108 (31.6)	50 (28.6)	58 (34.7)
Any negative emotion	79 (23.1)	39 (22.3)	40 (24.0)	84 (24.6)	44 (25.1)	40 (24.0)
Ate with family, friends, or colleagues	164 (48.0)	93 (53.1)	71 (42.5)	183 (53.5)	93 (53.1)	90 (53.9)
Ate away from home	174 (50.9)	98 (56.0)	76 (45.5)	181 (52.9)	92 (52.6)	89 (53.3)
Food was self-prepared	86 (25.1)	37 (21.1)	49 (29.3)	78 (22.8)	30 (17.1)	48 (28.7)
Screen activity immediately before eating	46 (13.5)	17 (9.7)	29 (17.4)	55 (16.1)	23 (13.1)	32 (19.2)
Exercise immediately before eating ^1^	8 (2.3)	5 (2.9)	3 (1.8)	21 (6.1)	13 (7.4)	8 (4.8)
**Postprandial glucose response**						
Premeal, mmol/L	—	—	—	5.9 (5.4–6.4)	5.9 (5.5–6.3)	6.0 (5.4–6.7)
Mean 0–120 min glucose, mmol/L	—	—	—	7.3 ± 1.3	7.4 ± 1.3	7.3 ± 1.3
Positive 0–120 min iAUC, mmol/L·min	—	—	—	137.2 (61.1–244.3)	153.2 (81.4–247.5)	128.4 (45.6–233.1)
Peak glucose, mmol/L	—	—	—	8.9 ± 1.8	8.9 ± 1.8	8.8 ± 1.9
Time to peak, min	—	—	—	57.7 ± 30.1	56.8 ± 28.5	58.6 ± 31.7

Values are median (IQR), mean ± SD, or No. (%). Male and female columns represent 175 pairs from 26 men and 167 pairs from 29 women, respectively. Percentages use pair denominators. Occasions may appear in both roles across pairs. Glucose responses refer to the current occasion; concentrations are in mmol/L. ^1^ Participants recorded context while logging food. Prior exercise and screen activity refer to the activity immediately before eating; exercise duration and intensity were unavailable. CGM, continuous glucose monitoring; GI, glycemic index; iAUC, incremental area under the curve; IQR, interquartile range; SD, standard deviation.

## Data Availability

The data presented in this study are available on request from the corresponding author due to privacy and ethical restrictions under the conditions of the Institutional Review Board approval. The CGMacros dataset used for the external comparison is openly available through PhysioNet at https://physionet.org/content/cgmacros/1.0.0/ (accessed on 27 July 2026).

## Supplementary Materials

The following supporting information can be downloaded at https://www.mdpi.com/article/10.3390/nu18213697/s1. Table S1: Interaction between intermeal interval and preceding-meal composition in relation to next-meal positive iAUC; Table S2: Associations of preceding-meal characteristics with next-meal positive iAUC in the CGMacros dataset; Table S3: Associations after additional adjustment for current-meal protein, total fat and fiber; Table S4: Associations after exclusion of participants with self-reported diabetes; Table S5: Variance inflation factors for the primary and current-meal macronutrient-adjusted models; Table S6: Associations after exclusion of pairs flagged for suspected unlogged eating; Table S7: Associations after additional adjustment for contextual variables; Table S8: Associations of liquid and solid sugar sources with next-meal positive iAUC; Checklist S1: STROBE and STROBE-nut reporting checklist.

## Abbreviations

The following abbreviations are used in this manuscript: BMI, body mass index; CGM, continuous glucose monitoring; CI, confidence interval; CV, coefficient of variation; eTRIP, eating Trigger Response Inhibition Program; GAD-2, 2-item Generalized Anxiety Disorder; GI, glycemic index; GL, glycemic load; HPB, Health Promotion Board; iAUC, incremental area under the curve; IMI, intermeal interval; IPAQ-SF, International Physical Activity Questionnaire–Short Form; IQR, interquartile range; PHQ-2, 2-item Patient Health Questionnaire; PPGR, postprandial glucose response; SD, standard deviation; SREBQ, Self-Regulation of Eating Behaviour Questionnaire; STROBE-nut, Strengthening the Reporting of Observational Studies in Epidemiology—Nutritional Epidemiology; TFEQ, Three-Factor Eating Questionnaire.
