# Supplementary material for: The Second-Meal Effect Under Free-Living Conditions: A Continuous Glucose Monitoring Study of Meal History and Postprandial Glycemia
Source: Nutrients. 2026 Nov 7;18(21):3697. doi: 10.3390/nu18213697 (PMC13765821; doi:10.3390/nu18213697)
Supplement: Supplementary file 1 [file nutrients-18-03697-s001.zip › Checklist_S1_STROBE-nut.pdf]

Checklist S1: STROBE and STROBE-nut reporting checklist

The second-meal effect under free-living conditions: A continuous glucose monitoring study of meal history and postprandial glycemia

STROBE reporting map

Item	Reporting topic	Manuscript location	Reported information or omission
1a–b	Design and balanced abstract	Abstract	Reported: abstract calls the study observational; structured summary retained.
2	Rationale	Introduction	Reported.
3	Objectives and hypotheses	Introduction, final paragraph	Objective reported. Prespecification of hypotheses is not stated.
4	Key design features	Study design and participants	Observational analysis within a three-arm trial described.
5	Setting and dates	Study design and participants	Singapore context reported. Recruitment and data-collection dates, sites and season specified.
6a	Eligibility, selection and follow-up	Study design and participants; Figure 1	Registered parent-trial inclusion and exclusion criteria and secondary-analysis pair eligibility are described.
6b	Matching of exposed and unexposed groups	Statistical analysis	No participant-matched groups described. Within-person models are used; Figure 3 matching is illustrative only.
7	Definitions of variables	Methods; Figure 2; Tables 1–2	Main exposures, outcome and adjustment factors described.
8	Data sources and measurement	Baseline characteristics; Dietary records and nutrient processing; CGM outcome; Statistical analysis	CGM, diet app, databases and baseline instruments described.
9	Bias control	Construction of consecutive eating-occasion pairs; Statistical analysis; Strengths and limitations	Non-overlap rules, fixed effects and adjustment reported; several sources of residual confounding and measurement error discussed.
10	Derivation of study size	Study design and participants; Figure 1; Participants and analytic eating-occasion pairs	Available-sample flow reported.
11	Handling quantitative variables	Methods; Figure 2	Nutrient densities, log transformation and within-person scaling described.
12a	Models and confounding	Statistical analysis; Figure 2 legend	Models, covariates, transformation and clustered bootstrap reported.

Item	Reporting topic	Manuscript location	Reported information or omission
12b	Interactions and subgroups	Statistical analysis; Relationship between preceding-meal factors and PPGR; Table S1	Exploratory IMI interactions reported in Table S1.
12c	Missing-data methods	Methods; Figure 1	Nutrient and CGM eligibility described. Missing covariates were handled by complete-case analysis with no imputation.
12d	Loss to follow-up	Study design and participants; Figure 1; Table 1	CGM sample attrition and coverage described.
12e	Sensitivity methods	Statistical analysis; Sensitivity analyses; Tables S3–S8	Current-meal nutrient adjustment, reported-diabetes exclusion, CGM-event exclusion, context and sugar-form sensitivities are reported in Tables S3–S8.
13a–c	Participant flow, exclusions and diagram	Study design and participants; Construction of consecutive eating-occasion pairs; Figure	Figure 1 reports the original 55-participant/342-pair within-day cohort. Exclusion of two reported diabetes histories is supplementary only (S4).
14a	Participant and exposure characteristics	Tables 1–2; Participants and analytic eating-occasion pairs	Reported overall. Exposures are continuous within-person variables, so there are no fixed exposed/unexposed groups.
14b	Missingness by variable	Tables 1–2; Figure 1	Reported overall
14c	Follow-up duration	Study design and participants; CGM outcome; Table 1	Ten-day monitoring specified; coverage and observed-day summaries provided.
15	Outcome summaries over observation	Tables 1–2; Figure 3	Glucose summaries and illustrative profiles reported. Continuous glucose outcome; no clinical event count is applicable.
16a	Estimates, precision and adjustment	Relationship between preceding-meal factors and PPGR; Figure 2; Table S2	Primary effects, CIs, P and BH q values reported in Figure 2; expanded current-macro model in S3 and diabetes exclusion in S4.
16b	Boundaries of categories	Statistical analysis; Figure 3	Primary exposures continuous. Lower/higher sugar examples described as extremes; explicit selection thresholds are not given.
16c	Translation of relative to absolute risk	Relationship between preceding-meal factors and PPGR; Figure 2	Not applicable to clinical risk: the outcome is transformed continuous iAUC, not an event risk.
17	Other analyses	Statistical analysis; Sensitivity analyses; Tables S3–S8	Sensitivity analyses and collinearity diagnostics are reported in Tables S3–S8.
18	Key findings	Discussion; Conclusions	Reported.
19	Bias and imprecision limitations	Strengths and limitations	Observational confounding, measurement error, precision and population limitations discussed; direction and magnitude of bias not quantified.

Item	Reporting topic	Manuscript location	Reported information or omission
20	Interpretation and multiplicity	Discussion; Conclusions	BH q values across 12 primary exposures; IMI, sugar and protein meet the primary threshold. Protein is sensitive to additional current-meal macronutrient adjustment; saturated fat is uncertain. Incomplete metabolic information and residual confounding are acknowledged.
21	Generalizability	Strengths and limitations	Generalizability beyond adults with overweight or obesity in Singapore is discussed.
22	Funding and funder role	Back matter, Funding	Funder and grant number reported; funder role specified.

STROBE nut extension

Item	Reporting topic	Manuscript location	Reported information or omission
nut-1	Dietary assessment identified	Abstract, Methods	Smartphone food recording stated.
nut-5	Context affecting diet	Study design and participants	Participants selected their own foods; no dietary plan was provided. Context was recorded while logging food. Visible CGM and potential behavioral reactivity are described.
nut-6	Nutritional selection criteria	Study design and participants	Parent-trial registered criteria are reported. Two self-reported diabetes cases remain in the primary cohort and are excluded only in S4. Metabolic-screening information was unavailable and glucose-lowering medication use was not recorded.
nut-7.1	Food and nutrient definitions	Dietary records and nutrient processing; Preceding-meal sugar and saturated fat	Twelve preceding-meal factors listed. Total sugars are not separated into added/free and intrinsic sources.
nut-7.2	Dietary indices and patterns	Dietary records and nutrient processing	Meal GL and weighted GI calculations described; no dietary-pattern score used.
nut-8.1	Dietary recording procedures	Dietary records and nutrient processing; Table 1	App photographs and descriptions reported.
nut-8.2	Composition databases and matching	Dietary records and nutrient processing	HPB database and GI fallback identified.
nut-8.3	Comparison with dietary requirements	Not applicable	No adequacy analysis against nutrient recommendations is reported.
nut-8.4	Nutritional biomarkers	CGM outcome	No biomarker used to quantify dietary intake. CGM is the glycemic outcome; confirm whether any dietary biomarker procedure is relevant.
nut-8.5	Non-dietary measurement and timing	Baseline characteristics; CGM outcome	Baseline instruments and CGM described.
nut-8.6	Validity and calibration	Dietary records and nutrient processing; Strengths and limitations	No study-specific validation of food recognition or nutrient assignment reported.
nut-9	Assessment bias handling	Methods; Strengths and limitations	The valid logging-day definition and its limitations are described. Suspected unlogged-eating CGM screening and exclusion analyses are reported in S6.
nut-11	Dietary categories and nonconsumers	Statistical analysis; Figure 3	Continuous primary exposures

Item	Reporting topic	Manuscript location	Reported information or omission
nut-12.1	Combining dietary data	Dietary records and nutrient processing	Meal GL sum and carbohydrate-weighted GI stated; recipe aggregation and supplement treatment not detailed.
nut-12.2	Energy adjustment and weighting	Statistical analysis; Figure 2 legend	Exposure-specific energy/covariate adjustment, nutrient percentages, fiber density and within-person scaling reported.
nut-12.3	Measurement-error correction	Statistical analysis; Strengths and limitations	No calibration-based correction described.
nut-13	Diet-related exclusions	Dietary records and nutrient processing; Figure 1	Meal-level counts reported. Participant exclusions specifically caused by missing/implausible diet data are not fully separated.
nut-14	Exposure distributions and denominators	Tables 1–2	Overall and preceding/current summaries supplied. Dietary summaries identify nutrient-complete meals; no exposure strata used.
nut-16	Supplement inclusion in intake	Dietary records and nutrient processing	Dietary supplements were not recorded or included in the nutrient estimates
nut-17	Sensitivity analyses and imputation	Statistical analysis; Sensitivity analyses; Tables S3–S8	Current-meal nutrient, diabetes, CGM-event, context and sugar-form sensitivities are reported in Tables S3–S8. No energy calibration or imputation was applied.
nut-19	Assessment limitations	Preceding-meal sugar and saturated fat; Strengths and limitations	Logging completeness, nutrient-source error, CGM/app feedback, context timing and metabolic-information limitations described.
nut-20	Nutritional relevance	Discussion; Conclusions	Second-meal interpretation and future implications given.
nut-22.1	Ethics and consent	Study design and participants; Ethical considerations; Consent to participate	IRB approval and written informed consent stated.
nut-22.2	Access to tools and data	Data availability	Request-based participant data and public CGMacros link supplied; access to collection tools/code not specified.

Items are paraphrased from the STROBE cohort checklist and Lachat et al., PLoS Medicine 2016;13:e1002036 (CC BY). The cohort framework is applied to the temporal meal-pair analysis; items concerning group matching, event risk and loss to follow-up are interpreted in that context.
