# Supplementary material for: The Second-Meal Effect Under Free-Living Conditions: A Continuous Glucose Monitoring Study of Meal History and Postprandial Glycemia
Source: Nutrients. 2026 Nov 7;18(21):3697. doi: 10.3390/nu18213697 (PMC13765821; doi:10.3390/nu18213697)
Supplement: Supplementary file 1 [file nutrients-18-03697-s001.zip › Supplementary_Tables_S1-S8.pdf]

Supplementary tables

Supplementary Tables S1 to S8

Table S1. Interaction between intermeal interval and preceding-meal composition in relation to next-meal positive iAUC.

Prior-meal exposure	Interaction, % (95% CI)	P interaction
Protein, % energy	+4.8% (−11.4 to +19.1)	0.507
Fat, % energy	+7.5% (−4.8 to +19.2)	0.210
Carbohydrate, % energy	−5.4% (−15.8 to +9.5)	0.383
Total sugar, % energy	−1.5% (−11.2 to +8.9)	0.704
Saturated fat, % energy	+7.2% (−3.3 to +17.5)	0.175
Weighted GI	+8.8% (−8.9 to +29.1)	0.316
Glycemic load	+2.4% (−8.6 to +24.5)	0.757
Fiber density	−1.9% (−11.0 to +11.4)	0.812

Values are percentage interaction effects (95% CIs). CIs and two-sided P values were estimated using 2,000 participant-cluster bootstrap resamples. P values were not adjusted for multiple testing.

Interaction terms were products of within-person standardized IMI and composition; both main effects were included. Models included participant fixed effects and adjusted for current-meal GL and energy intake, premeal glucose, study day, clock hour, weekend status and exposure-specific preceding-meal covariates. Analyses included 342 pairs from 55 participants, except weighted GI (304 pairs from 53 participants; preceding-meal carbohydrate intake ≥10 g).

CI, confidence interval; GI, glycemic index; GL, glycemic load; iAUC, incremental area under the curve; IMI, intermeal interval; SD, standard deviation.

Table S2. Associations of preceding-meal characteristics with next-meal positive iAUC in the CGMacros dataset.

Factor	Pairs	Within-SD unit	% per within-SD (95% CI)	P value
Intermeal interval	615	1.15 h	+33.6% (+13.3 to +54.7)	0.001
Prior meal energy	615	247 kcal	−33.4% (−42.6 to −21.9)	0.001
Prior fat %	615	13.1 %E	+28.0% (+11.0 to +46.2)	0.001
Prior protein %	615	13.5 %E	−10.3% (−18.5 to +3.3)	0.166
Prior carbohydrate %	615	13.9 %E	−6.8% (−17.2 to +1.3)	0.093
Prior fiber density	614	48.5 g/1000 kcal	−7.1% (−11.3 to +78.4)	0.650

Values are percentage differences (95% CIs) in positive iAUC + 1 per within-person SD. Models used ln(1 + positive iAUC) over 0–120 min. CIs and two-sided P values were estimated using 2,000 participant-cluster bootstrap resamples. P values were not adjusted for multiple testing.

Models included participant fixed effects and adjusted for current-meal carbohydrate and energy intake, premeal glucose, study day, clock hour and weekend status. Models of factors other than IMI additionally adjusted for log-transformed IMI. Preceding-meal energy was included for composition exposures; protein and fat models additionally included preceding-meal fat and protein percentages, respectively. The IMI model included preceding-meal energy, protein percentage and fat percentage.

CI, confidence interval; iAUC, incremental area under the curve; IMI, intermeal interval; SD, standard deviation.

Table S3. Associations after additional adjustment for current-meal protein, total fat and fiber.

Preceding-meal factor	Pairs / participants	Primary model, % (95% CI)	Additional adjustment, % (95% CI)	P value	q value
Intermeal interval	342/55	+29.1 (+15.0 to +48.8)	+27.4 (+12.5 to +47.7)	0.001	0.006
Meal duration	326/55	+4.3 (−5.4 to +13.9)	+3.8 (−5.2 to +14.1)	0.398	0.530
Meal energy	342/55	−2.8 (−14.6 to +10.5)	−1.3 (−13.5 to +11.9)	0.841	0.888
Glycaemic load	342/55	−10.1 (−26.4 to +10.5)	−8.6 (−24.7 to +11.4)	0.385	0.530
Prior meal positive iAUC	340/55	−3.7 (−13.0 to +6.6)	−3.8 (−13.1 to +7.2)	0.456	0.547
Weighted GI	304/53	−8.3 (−22.1 to +7.2)	−7.9 (−20.5 to +6.1)	0.234	0.401
Total sugar % energy	342/55	+29.6 (+16.9 to +45.2)	+29.3 (+14.9 to +43.9)	0.001	0.006
Fibre density	342/55	+6.9 (−2.1 to +22.4)	+7.4 (−2.3 to +25.6)	0.160	0.320
Carbohydrate % energy	342/55	+8.1 (−2.8 to +20.3)	+8.3 (−2.7 to +20.4)	0.131	0.314
Saturated fat % energy	342/55	+14.8 (−0.8 to +35.2)	+16.0 (+0.5 to +35.2)	0.048	0.144
Fat % energy	342/55	+0.3 (−9.1 to +10.6)	−0.4 (−9.5 to +9.9)	0.888	0.888
Protein % energy	342/55	−13.9 (−23.1 to −4.2)	−13.4 (−22.4 to −3.5)	0.013	0.052

Values are percentage differences (95% CIs) in positive iAUC + 1 per within-person SD. CIs and two-sided P values were estimated using 2,000 participant-cluster bootstrap resamples. Benjamini–Hochberg q values were calculated across 12 exposures. Sample sizes, P values and q values refer to the additionally adjusted models.

Participant fixed-effects models used the primary covariate specification and exposure SDs, with additional adjustment for current-meal protein and total fat (% energy) and fiber density (g/1,000 kcal). Sample sizes reflect exposure-specific complete-case eligibility.

CI, confidence interval; GI, glycemic index; iAUC, incremental area under the curve; SD, standard deviation.

Table S4. Associations after exclusion of participants with self-reported diabetes.

Preceding-meal factor	Pairs / participants	Primary model, % (95% CI)	Excluding diabetes, % (95% CI)	P value	q value
Intermeal interval	330/53	+29.1 (+15.0 to +48.8)	+29.3 (+14.1 to +50.6)	0.001	0.006
Meal duration	315/53	+4.3 (−5.4 to +13.9)	+4.6 (−5.2 to +14.4)	0.343	0.457
Meal energy	330/53	−2.8 (−14.6 to +10.5)	−1.4 (−13.3 to +12.1)	0.771	0.841
Glycaemic load	330/53	−10.1 (−26.4 to +10.5)	−6.9 (−21.8 to +12.3)	0.457	0.548
Prior meal positive iAUC	328/53	−3.7 (−13.0 to +6.6)	−7.1 (−15.6 to +2.1)	0.151	0.302
Weighted GI	295/51	−8.3 (−22.1 to +7.2)	−7.6 (−21.2 to +7.0)	0.282	0.423
Total sugar % energy	330/53	+29.6 (+16.9 to +45.2)	+28.6 (+15.3 to +43.7)	0.001	0.006
Fibre density	330/53	+6.9 (−2.1 to +22.4)	+5.6 (−3.7 to +25.6)	0.263	0.423
Carbohydrate % energy	330/53	+8.1 (−2.8 to +20.3)	+9.2 (−2.2 to +21.9)	0.103	0.247
Saturated fat % energy	330/53	+14.8 (−0.8 to +35.2)	+17.7 (+0.9 to +37.9)	0.037	0.111
Fat % energy	330/53	+0.3 (−9.1 to +10.6)	−0.4 (−9.4 to +9.3)	0.914	0.914
Protein % energy	330/53	−13.9 (−23.1 to −4.2)	−15.2 (−24.0 to −6.1)	0.003	0.012

Values are percentage differences (95% CIs) in positive iAUC + 1 per within-person SD. CIs and two-sided P values were estimated using 2,000 participant-cluster bootstrap resamples. Benjamini–Hochberg q values were calculated across 12 exposures. Sample sizes, P values and q values refer to the analysis excluding participants with self-reported diabetes.

Two participants with self-reported diabetes were excluded, retaining 330 pairs from 53 participants before exposure-specific restrictions. Models used the primary covariate specification and exposure SDs. Metabolic screening information was unavailable for this secondary analysis, and glucose-lowering medication use was not recorded.

CI, confidence interval; GI, glycemic index; iAUC, incremental area under the curve; SD, standard deviation.

Table S5. Variance inflation factors for the primary and current-meal macronutrient-adjusted models.

A. Preceding-meal exposures

Predictor	Primary model	Additional current-meal macronutrient adjustment
Intermeal interval	1.13	1.17
Meal duration	1.07	1.08
Meal energy	1.23	1.28
Glycemic load	2.69	2.75
Preceding-meal glucose response (iAUC)	1.14	1.15
Weighted GI	1.15	1.20
Total sugar (% energy)	1.80	1.80
Fiber density	1.06	1.07
Carbohydrate (% energy)	1.26	1.28
Saturated fat (% energy)	2.72	2.75
Total fat (% energy)	1.21	1.23
Protein (% energy)	1.15	1.16

B. Adjustment covariates (maximum VIF)

Predictor	Primary model	Additional current-meal macronutrient adjustment
Current-meal carbohydrate	3.43	6.11
Current-meal energy	3.61	5.75
Current-meal protein	Not included	1.35
Current-meal total fat	Not included	1.69
Current-meal fiber	Not included	1.10
Log intermeal interval	1.21	1.26
Clock time	1.23	1.24

VIFs were calculated from within-person centered predictor matrices. Panel B reports the maximum VIF across the exposure-specific models. The maximum VIF for other adjustment covariates was 4.52 in the primary models and 4.53 in the additionally adjusted models.

Additional adjustment comprised current-meal protein and total fat (% energy) and fiber density (g/1,000 kcal). Log-transformed IMI was included only in models of non-IMI exposures.

GI, glycemic index; iAUC, incremental area under the curve; IMI, intermeal interval; VIF, variance inflation factor.

Table S6. Associations after exclusion of pairs flagged for suspected unlogged eating.

Factor	Pairs / participants	Effect, % (95% CI)	P value
Intermeal interval	305/55	+32.5 (+14.9 to +56.6)	0.001
Meal duration	292/55	+4.8 (−5.1 to +15.5)	0.332
Meal energy	305/55	−3.3 (−16.0 to +11.2)	0.619
Glycaemic load	305/55	−8.5 (−26.5 to +15.3)	0.449
Prior meal positive iAUC	303/55	−7.8 (−15.8 to +0.5)	0.068
Weighted GI	271/53	−7.0 (−21.7 to +9.2)	0.366
Total sugar % energy	305/55	+27.6 (+13.1 to +45.1)	0.001
Fibre density	305/55	+5.2 (−4.1 to +19.9)	0.287
Carbohydrate % energy	305/55	+8.6 (−1.7 to +22.3)	0.113
Saturated fat % energy	305/55	+10.1 (−4.4 to +32.2)	0.187
Fat % energy	305/55	−0.1 (−10.1 to +9.9)	0.967
Protein % energy	305/55	−15.9 (−26.2 to −5.2)	0.006

Values are percentage differences (95% CIs) in positive iAUC + 1 per within-person SD. Models used the primary covariate specification and exposure SDs. CIs and two-sided P values were estimated using 2,000 participant-cluster bootstrap resamples. P values were not adjusted for multiple testing.

CGM screening excluded the first 180 min after the preceding meal and the final 30 min before the current meal. Intervals required ≥45 min of remaining observation and ≥80% CGM coverage. Glucose readings were summarized in 5-min median bins and smoothed using a centered three-bin median. Suspected events were defined as a rise of ≥1.5 mmol/L within 30 min that persisted for ≥10 min above half the rise. The current postprandial response window was not screened.

Of 342 intervals, 204 met the screening criteria and 37 were flagged. Exclusion retained 305 pairs from 55 participants before exposure-specific complete-case restrictions, including 138 intervals that could not be screened; these were not classified as free of suspected events. In the subset meeting screening criteria after exclusion (167 pairs from 49 participants), the sugar estimate was less precise (+15.7%; 95% CI, −2.2 to +35.8; P = 0.099). Flagged CGM rises do not confirm eating.

CGM, continuous glucose monitoring; CI, confidence interval; iAUC, incremental area under the curve; SD, standard deviation.

Table S7. Associations after additional adjustment for contextual variables.

Factor	Pairs / participants	Effect, % (95% CI)	P value
Intermeal interval	283/53	+34.9 (+15.9 to +62.8)	0.001
Meal duration	269/53	+7.9 (−6.5 to +22.6)	0.292
Meal energy	283/53	+1.9 (−10.2 to +15.0)	0.800
Glycaemic load	283/53	−7.8 (−26.1 to +12.5)	0.447
Prior meal positive iAUC	283/53	−9.8 (−19.2 to +0.1)	0.054
Weighted GI	250/51	−5.2 (−23.1 to +11.2)	0.454
Total sugar % energy	283/53	+28.9 (+8.1 to +52.6)	0.006
Fibre density	283/53	−0.4 (−8.6 to +11.8)	0.899
Carbohydrate % energy	283/53	+5.2 (−8.3 to +17.7)	0.492
Saturated fat % energy	283/53	+16.1 (−6.6 to +39.4)	0.191
Fat % energy	283/53	+3.7 (−6.9 to +16.4)	0.538
Protein % energy	283/53	−13.7 (−24.5 to −1.6)	0.026

Values are percentage differences (95% CIs) in positive iAUC + 1 per within-person SD. Models used the primary covariate specification and exposure SDs. CIs and two-sided P values were estimated using 2,000 participant-cluster bootstrap resamples. P values were not adjusted for multiple testing.

Participants recorded context while logging food. Models additionally adjusted for self-reported hunger, negative emotions, social company, location, screen activity and prior exercise for both occasions. The timing-qualified cohort comprised 283 pairs from 53 participants with all selected responses recorded within 60 min of both occasions; sample sizes reflect exposure-specific complete-case eligibility. Exercise duration and intensity were unavailable, and emotion ratings did not directly measure emotional eating.

Timing, self-report validity and selected-subset representativeness limit interpretation. Current-meal context may follow the preceding exposure; these sensitivities do not identify causal effects.

CI, confidence interval; GI, glycemic index; iAUC, incremental area under the curve; SD, standard deviation.

Table S8. Associations of liquid and solid sugar sources with next-meal positive iAUC.

Sugar source	Effect, % (95% CI)	P value	q value	SD, percentage points
Liquid sugar	+22.1 (+11.2 to +36.0)	0.001	0.001	16.95
Solid sugar	+24.1 (+11.4 to +40.1)	0.001	0.001	14.68

Values are percentage differences (95% CIs) in positive iAUC + 1 per within-person SD. CIs and two-sided P values were estimated using 2,000 participant-cluster bootstrap resamples. Benjamini–Hochberg q values were calculated across the two sugar-source estimates.

Original logged food names classified 479 preceding food entries as 99 liquid, 372 solid/semi-solid and eight mixed/unspecified entries. Seven pairs with unseparated sugar from ambiguous entries were excluded, leaving 335 pairs from 55 participants. Yoghurt, soup and ice cream were grouped with solids/semi-solids. Sugar quantities were taken from the existing nutrient records.

Liquid and solid sugar shares were mutually adjusted with the primary covariates and preceding-meal covariates. Source-specific SDs differ, so the estimates do not directly compare liquid with solid sugar. Fructose dose was not measured. Food-name classification and nutrient mapping remain uncertain.

CI, confidence interval; iAUC, incremental area under the curve; SD, standard deviation.
